# Extracellular pneumolysin enhances the activation of cytosolic pattern-recognition receptor NOD2 through its pore-forming activity

**DOI:** 10.1128/spectrum.00249-26

**Published:** 2026-07-14

**Authors:** Hisanori Domon, Satoru Hirayama, Toshihito Isono, Tomoki Maekawa, Yutaka Terao

**Affiliations:** 1 Division of Microbiology and Infectious Diseases, Niigata University Graduate School of Medicine, Dentistry and Health Sciences 157848, Niigata, Japan; 2 Center for Advanced Oral Science, Niigata University, Graduate School of Medicine, Dentistry and Health Sciences 157848, Niigata, Japan; Cinvestav Unidad Zacatenco, Mexico City, Mexico

**Keywords:** *Streptococcus pneumoniae*, pneumolysin, pore-forming toxin, NOD2, innate immunity

## Abstract

*Streptococcus pneumoniae* is the leading cause of community-acquired pneumonia, meningitis, and sepsis. This bacterium produces pneumolysin, a cholesterol-dependent cytolysin that forms oligomeric transmembrane pores in the host cells. Although pneumolysin is known to exhibit proinflammatory properties, the mechanisms by which it activates innate immune responses remain to be investigated. Here, we show that extracellular pneumolysin enhances the activation of nucleotide-binding oligomerization domain 2 (NOD2), a cytosolic receptor that recognizes bacterial peptidoglycans. Experiments using HEK-Blue cell lines expressing specific pattern recognition receptors revealed that pneumolysin does not directly activate Toll-like receptors or other innate immune receptors. In contrast, our findings suggest that pneumolysin-generated membrane pores may facilitate the cytosolic entry of peptidoglycan, thereby contributing to an elevated activation of NOD2. Furthermore, pneumolysin enhances NOD1 activation in cells stimulated with a NOD1 ligand, suggesting a broader role for pore-forming toxins in innate immunity. These findings shed light on *S. pneumoniae*-modulated immune modulation and highlight toxin-induced immune pathways as potential therapeutic targets.

**IMPORTANCE:**

The mechanisms by which the pneumococcal pore-forming toxin pneumolysin activates innate immune responses have not been fully understood. Specifically, it remains unclear whether pneumolysin is directly sensed by Toll-like receptor 4 (TLR4) or activates the NLRP3 inflammasome. Here, we show that pneumolysin is not a direct ligand for pattern-recognition receptors. Instead, pneumolysin forms membrane pores that increase plasma membrane permeabilization, thereby amplifying innate immune signaling through multiple pathways. These pores may provide a route for the cytosolic entry of pneumococcal peptidoglycan, contributing to enhanced activation of the cytosolic receptor nucleotide-binding oligomerization domain 2 (NOD2). Additionally, membrane pores may promote the extracellular release of damage-associated molecular patterns, such as high mobility group box 1 (HMGB1), providing a mechanistic explanation for previously reported pneumolysin-induced TLR4 activation. Furthermore, pore-induced ion efflux provides a framework to explain previously reported NLRP3 inflammasome activation. Together, our findings establish membrane permeabilization as a central mechanism by which pneumolysin modulates innate immune sensing during pneumococcal infection.

## INTRODUCTION

*Streptococcus pneumoniae*, also known as pneumococcus, is the leading cause of community-acquired pneumonia, otitis media, and invasive pneumococcal diseases such as sepsis and meningitis. This bacterium is also the main cause of morbidity and mortality among children younger than 5 years and adults older than 70 years (1). The Global Burden of Disease Study 2021 showed that an estimated 98 million people developed pneumococcal pneumonia worldwide, resulting in approximately 500,000 deaths in 2021 (2). Additionally, it has been reported that the total estimated annual cost of pneumococcal diseases is approximately $8 billion in the United States (3) and is expected to rise due to the growing aging population and increasing prevalence of antibiotic-resistant pneumococci.

In the early stages of infection or colonization, *S. pneumoniae* releases various proinflammatory molecules and virulence factors, activating the host’s innate immune response via pattern recognition receptors (PRRs). Different classes of PRRs have been identified, including Toll-like receptors (TLRs), nucleotide-binding oligomerization domain (NOD)-like receptors, RIG-I-like receptors, C-type lectin receptors, and cytosolic DNA sensors (4). Among these, TLR2, TLR3, TLR9, and NOD2 recognize pneumococcal lipoproteins/lipopeptides, RNA, genomic DNA, and peptidoglycan, respectively (5–8). Additionally, several studies have shown that pneumolysin (PLY), a pneumococcal pore-forming toxin, is recognized by TLR4 (9–11). These PRR/ligand interactions trigger the activation of transcription factors, such as NF-κB, resulting in the production of proinflammatory cytokines and subsequent neutrophil infiltration into the infection site.

PLY is a key virulence factor of *S. pneumoniae* that binds to cholesterol-rich cell membranes and forms ring-shaped oligomers, leading to host cell death (12). Although PLY is an intracellular protein, it is released predominantly during pneumococcal autolysis, which is a distinctive feature of *S. pneumoniae* (13). Once released, PLY exhibits cytotoxicity toward various cell types, including alveolar epithelial cells (14), neutrophils (15), and monocytes/macrophages (16). Neutrophils are particularly vulnerable to PLY-induced cell death. Subsequently, dead neutrophils release neutrophil elastase, destroying the host tissue (15). Although the cytotoxic effect of PLY has been considered crucial in the pathogenesis of pneumococcal diseases, García-Suárez et al. reported that, in a murine model of pneumococcal pneumonia, lung injury may be attributable to the proinflammatory activity rather than the cytotoxic effect of PLY (17). However, the PRRs that recognize PLY remain a subject of debate (18). Since 2003, when Malley et al. first reported that TLR4 mediates the host recognition of PLY (9), there have been several studies using murine bone marrow-derived dendritic cells and RAW 264.7 macrophages that have provided further evidence indicating a role for TLR4 in PLY-induced proinflammatory responses (10, 11). In contrast, other studies have reported that PLY activates the NLRP3 inflammasome and promotes proinflammatory cytokines independently of TLR4 (19, 20). These discrepancies highlight the need for further investigations to provide a more comprehensive understanding of PLY-induced innate immune activation.

In this study, we used endotoxin-free recombinant PLY (rPLY) to assess its proinflammatory activity against human embryonic kidney 293 (HEK) cells stably transfected with individual PRRs. Additionally, we investigated the synergistic effects of PLY and various PRR ligands on the production of proinflammatory cytokines in these cells and in the human monocytic cell line THP-1. Differences in proinflammatory activity between the wild-type pneumococcal strain and the isogenic *ply*-negative mutant strain were also analyzed.

## RESULTS

### PLY enhances activation of cytosolic receptors NOD1 and NOD2 without activating TLR4

**Fig 1:**
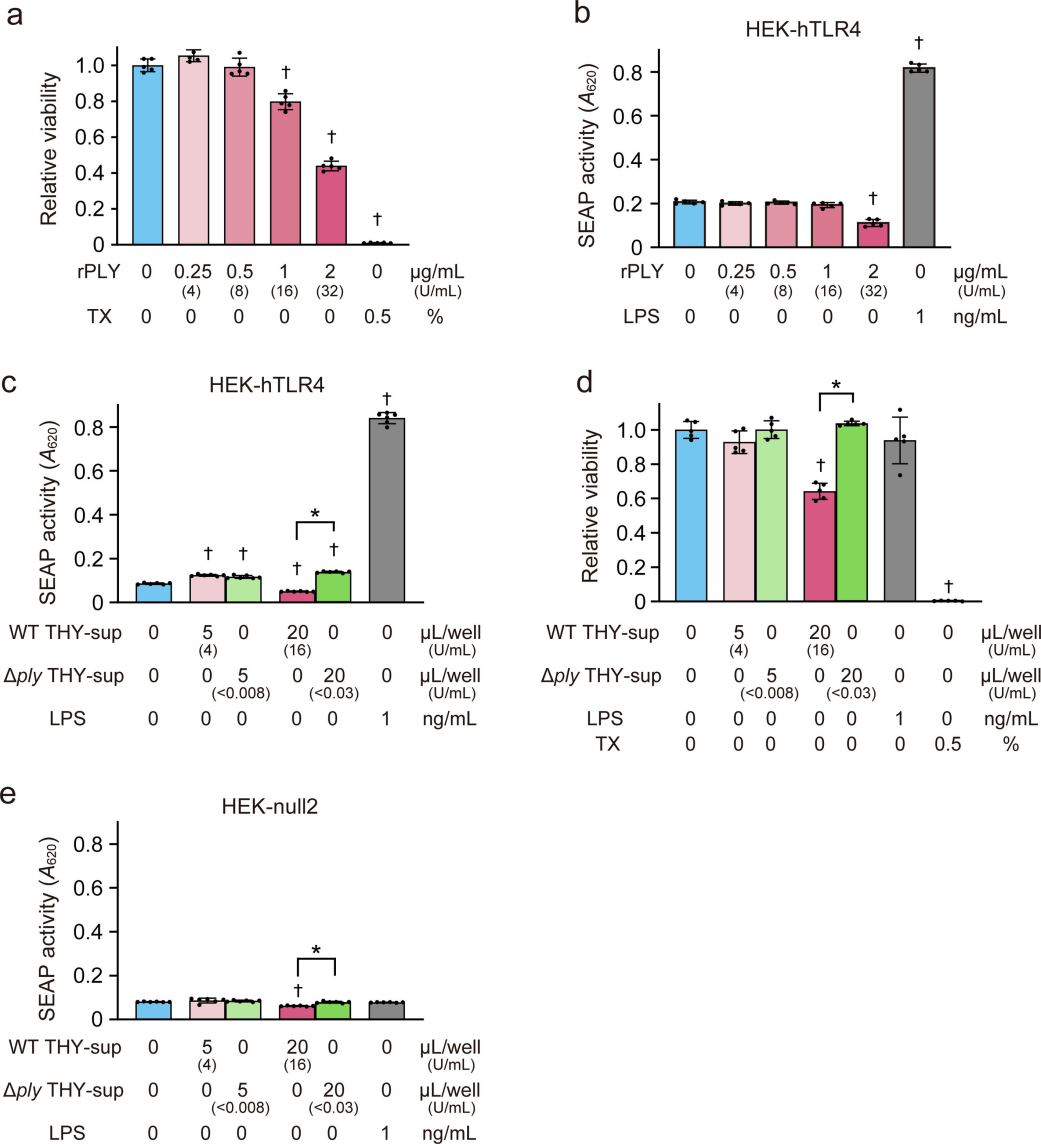
PLY does not activate Toll-like receptor 4 in HEK-Blue cells expressing human Toll-like receptor 4. (**a and b**) HEK-Blue cells expressing human TLR4 (HEK-hTLR4) were exposed to various concentrations of recombinant PLY (rPLY) for 16 h. (**a**) Cell viability assay was performed using 3-(4,5-dimethylthiazol-2-yl)-2,5-diphenyltetrazolium bromide (MTT). (**b**) SEAP levels in the culture supernatant were quantified using spectrophotometry at 620 nm. (**c and d**) HEK-hTLR4 were exposed to supernatant (sup) from *S. pneumoniae* wild-type strain D39 (WT) or Δ*ply* grown in Todd Hewitt yeast extract broth (THY) for 16 h. (**c**) SEAP levels in the culture supernatant were quantified using spectrophotometry. (**d**) Cell viability was assessed using the MTT assay. (**e**) HEK-null2 negative control cells were exposed to supernatant from *S. pneumoniae* wild-type strain D39 or Δ*ply* cultures for 16 h, followed by SEAP activity assay. Data represent the mean ± SD of quintuplicate or sextuplicate experiments and were evaluated using a one-way analysis of variance with (**a and b**) Dunnett’s or (**c and d**) Tukey’s multiple comparisons test. † indicates a significant difference compared with the control group (light blue) at *P* < 0.05. * indicates a significant difference between the indicated groups at *P* < 0.05. hTLR, human Toll-like receptor; rPLY, recombinant pneumolysin; SD, standard deviation; SEAP, secreted alkaline phosphatase; Sup, supernatant; THY, Todd Hewitt yeast extract broth; TX, Triton X-100.

The PRR-transfected HEK-Blue cell line used in this study expressed an inducible reporter gene encoding secreted embryonic alkaline phosphatase (SEAP), which is activated upon PRR-dependent NF-κB signaling, resulting in increased SEAP activity in cell culture media. Therefore, the activation of PRRs can be examined by measuring SEAP activity. We examined the rPLY cytotoxicity in HEK-Blue cells expressing human TLR4 (HEK-hTLR4). Figure 1a shows that ≥1 µg/mL rPLY significantly decreased the viability of the cells. SEAP activity assay using cell culture supernatant from rPLY-treated HEK-hTLR4 showed that ≤1 µg/mL rPLY did not significantly increase SEAP secretion into the supernatant compared to untreated control, whereas 2 μg/mL rPLY significantly decreased SEAP activity (Fig. 1b). These findings suggest that rPLY induced cytotoxicity without activating the TLR4/NF-κB pathway. HEK-hTLR4 was also incubated with the pneumococcal supernatant from wild-type (WT) strain D39 or a *ply*-isogenic mutant strain (Δ*ply*). As a result, culture supernatants of both WT and Δ*ply* (5 μL each, added to 200 μL cell culture medium) slightly but significantly increased SEAP activity compared to the untreated control (Fig. 1c). No significant difference was observed between the WT and Δ*ply* groups, suggesting that PLY in the culture supernatant of WT does not activate TLR4. However, 20 μL culture supernatants of WT significantly decreased SEAP activity compared to the untreated control. In contrast, 20 μL culture supernatants of Δ*ply* increased SEAP activity in HEK-hTLR4 (Fig. 1c). Figure 1d shows that only 20 μL culture supernatants of WT significantly decreased the cell viability, suggesting that PLY in the culture supernatants of WT (20 μL) induced cytotoxicity. Neither the culture supernatants of WT nor Δ*ply* increased SEAP activity in the culture supernatant of HEK-null2 negative control cells (Fig. 1e) that were transfected with the SEAP reporter gene alone. Therefore, these results indicate the PLY-independent activation of TLR4 by *S. pneumoniae*. We previously reported that pneumococcal DNA-binding proteins activate TLR4 signaling (21). Taken together, our data suggest that neither rPLY used in this study nor PLY in pneumococcal supernatant activates the TLR4/NF-κB pathway.

**Fig 2:**
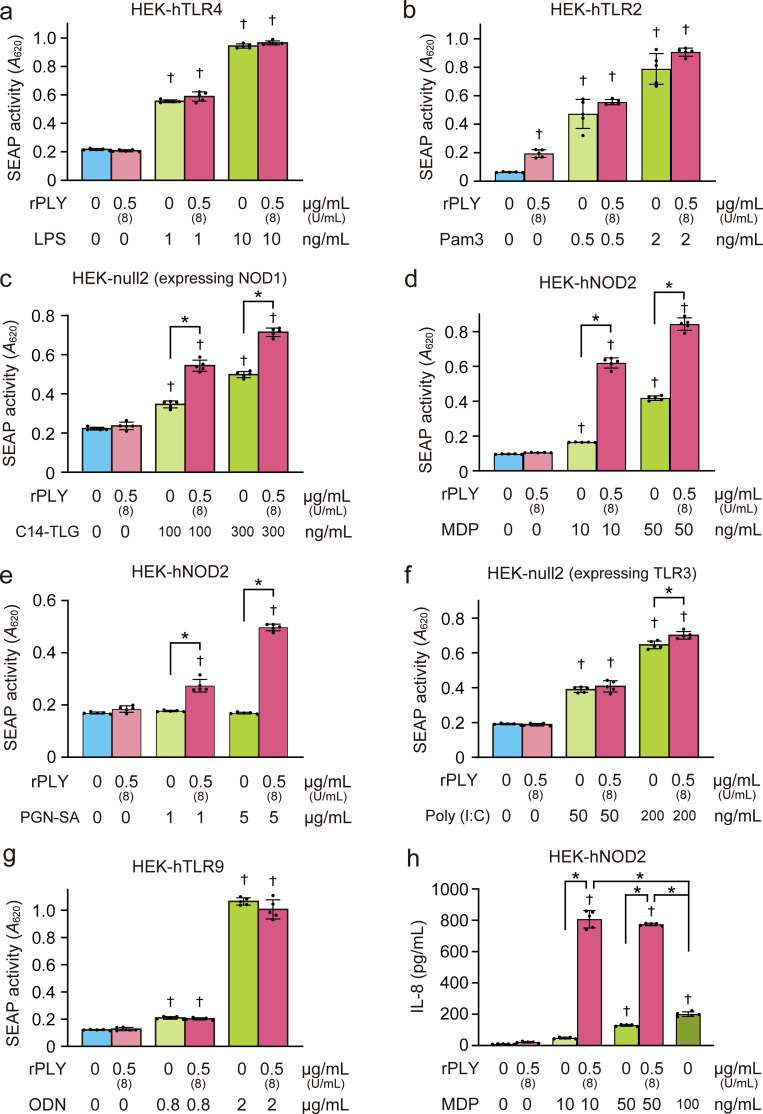
Recombinant PLY significantly enhances both NOD1 ligand- and NOD2 ligand-induced NF-κB activation in HEK-Blue cells. (**a**) HEK-hTLR4 cells were stimulated with LPS in the presence or absence of rPLY for 16 h, followed by SEAP activity assay. (**b**) HEK-hTLR2 cells were stimulated with Pam3CSK4 (Pam3) in the presence or absence of rPLY for 14 h, followed by SEAP activity assay. (**c**) HEK-null2 cells were stimulated with C14-Tri-LAN-Gly (C14-TLG) in the presence or absence of rPLY for 16 h, followed by SEAP activity assay. (**d**) HEK-hNOD2 cells were stimulated with muramyl dipeptide (MDP) in the presence or absence of rPLY for 14 h, followed by SEAP activity assay. (**e**) HEK-hNOD2 cells were stimulated with peptidoglycan from *Staphylococcus aureus* (PGN-SA) in the presence or absence of rPLY for 14 h, followed by SEAP activity assay. (**f**) HEK-null2 cells were stimulated with polyinosinic:polycytidylic acid [poly(I:C)] in the presence or absence of rPLY for 16 h, followed by SEAP activity assay. (**g**) HEK-hTLR9 cells were stimulated with CpG oligodeoxynucleotide 2006 (ODN) in the presence or absence of rPLY for 18 h, followed by SEAP activity assay. (**h**) HEK-hNOD2 cells were stimulated with MDP in the presence or absence of rPLY for 24 h. IL-8 levels in the culture supernatant were measured using ELISA. Data represent the mean ± SD of quintuplicate experiments and were evaluated using a one-way analysis of variance with Tukey’s multiple comparisons test. † indicates a significant difference compared with the control group (light blue) at *P* < 0.05. * indicates a significant difference between the indicated groups at *P* < 0.05. C14-TLG, C14-Tri-LAN-Gly; hTLR, human Toll-like receptor; MDP, muramyl dipeptide; NOD2, nucleotide-binding oligomerization domain-containing protein 2; ODN, CpG oligodeoxynucleotide 2006; Pam3, Pam3CSK4; PGN-SA, peptidoglycan from *S. aureus*; poly(I:C), polyinosinic:polycytidylic acid; rPLY, recombinant pneumolysin; SD, standard deviation; SEAP, secreted alkaline phosphatase.

A previous study demonstrated that rPLY synergizes with heat-killed *S. pneumoniae* to enhance the production of proinflammatory cytokines by dendritic cells (19). Additionally, *S. pneumoniae* activates TLR2, TLR3, TLR4, TLR9, and NOD2, resulting in NF-κB-dependent production of proinflammatory cytokines (6, 22). Therefore, we investigated whether rPLY at sublytic concentration (0.5 μg/mL) enhances the NF-κB activation by these PRRs using HEK-Blue cell lines. Figure 2a shows that 0.5 μg/mL rPLY did not significantly enhance lipopolysaccharide (LPS; TLR4 ligand)-induced SEAP production in HEK-hTLR4 cells. Although it was confirmed that rPLY does not activate or enhance TLR4 signaling and is therefore endotoxin-free, we found that rPLY induced SEAP production in HEK-hTLR2 cells. However, a previous study refuted this interaction between PLY and TLR2 (9). Therefore, we suspected lipoprotein contamination in rPLY and treated it with lipoprotein lipase (LPL) for lipoprotein digestion using previously reported methods (23). LPL treatment substantially decreased the TLR2-stimulatory activity of both rPLY and Pam3CSK4 (TLR2/TLR1 ligand) (Fig. S1). Next, we confirmed the reproducibility of the results shown in Fig. 1a, b, and 2a using LPL-treated rPLY. Therefore, subsequent experiments were performed using the LPL-treated rPLY. Although rPLY slightly induced SEAP production in HEK-TLR2 cells (Fig. 2b), which may be due to the residual presence of incompletely degraded lipoproteins (Fig. S1), it did not significantly amplify Pam3CSK4-induced SEAP production. However, rPLY significantly enhanced SEAP production induced by C14-Tri-LAN-Gly (C14-TLG; NOD1 ligand) and muramyl dipeptide (MDP; NOD2 ligand) in HEK-null2 cells, which express endogenous NOD1 and TLR3, and in HEK-hNOD2 cells, respectively (Fig. 2c and d). Ratner et al. also reported that peptidoglycan from *Haemophilus influenzae* synergizes with PLY and activates NOD1 (24). Next, we exposed HEK-hNOD2 cells to rPLY in the presence or absence of *Staphylococcus aureus* peptidoglycan (PGN-SA; NOD2 ligand [25]) and found that stimulation with a combination of rPLY and PGN-SA significantly elevated SEAP levels in the culture supernatant of the cells compared to stimulation with PGN-SA alone (Fig. 2e). Because NOD1 and NOD2 are intracellular cytosolic receptors (26), we hypothesized that rPLY would also amplify the activation of TLR3 and TLR9, which are both expressed in the endosome (27). However, rPLY exerted only a diminished effect on SEAP production in polyinosinic:polycytidylic acid [poly(I:C); TLR3 ligand]-treated HEK-null2 cells and CpG oligodeoxynucleotide 2006 (CpG ODN; TLR9 ligand)-treated HEK-hTLR9 cells (Fig. 2f and g). Figure 2h shows that rPLY also significantly enhanced MDP-induced interleukin (IL)-8 levels in the culture supernatant of HEK-hNOD2 cells. Additionally, IL-8 levels in the supernatants of cells treated with a combination of rPLY and 10–50 ng/mL MDP were significantly higher than those in cells treated with 100 ng/mL MDP alone. Neither stimulation with TLR stimulants (LPS, Pam3CSK4, MDP, PGN-SA, or CpG ODN) alone nor in combination with rPLY caused a notable increase in SEAP activity in the culture supernatant of HEK-null2 negative control cells (Fig. S2a–e). These findings suggest that rPLY can enhance innate immune signaling via the cytosolic receptors NOD1 and NOD2.

### Only pore-forming rPLY can enhance NOD1/NF-κB and NOD2/NF-κB activation

**Fig 3:**
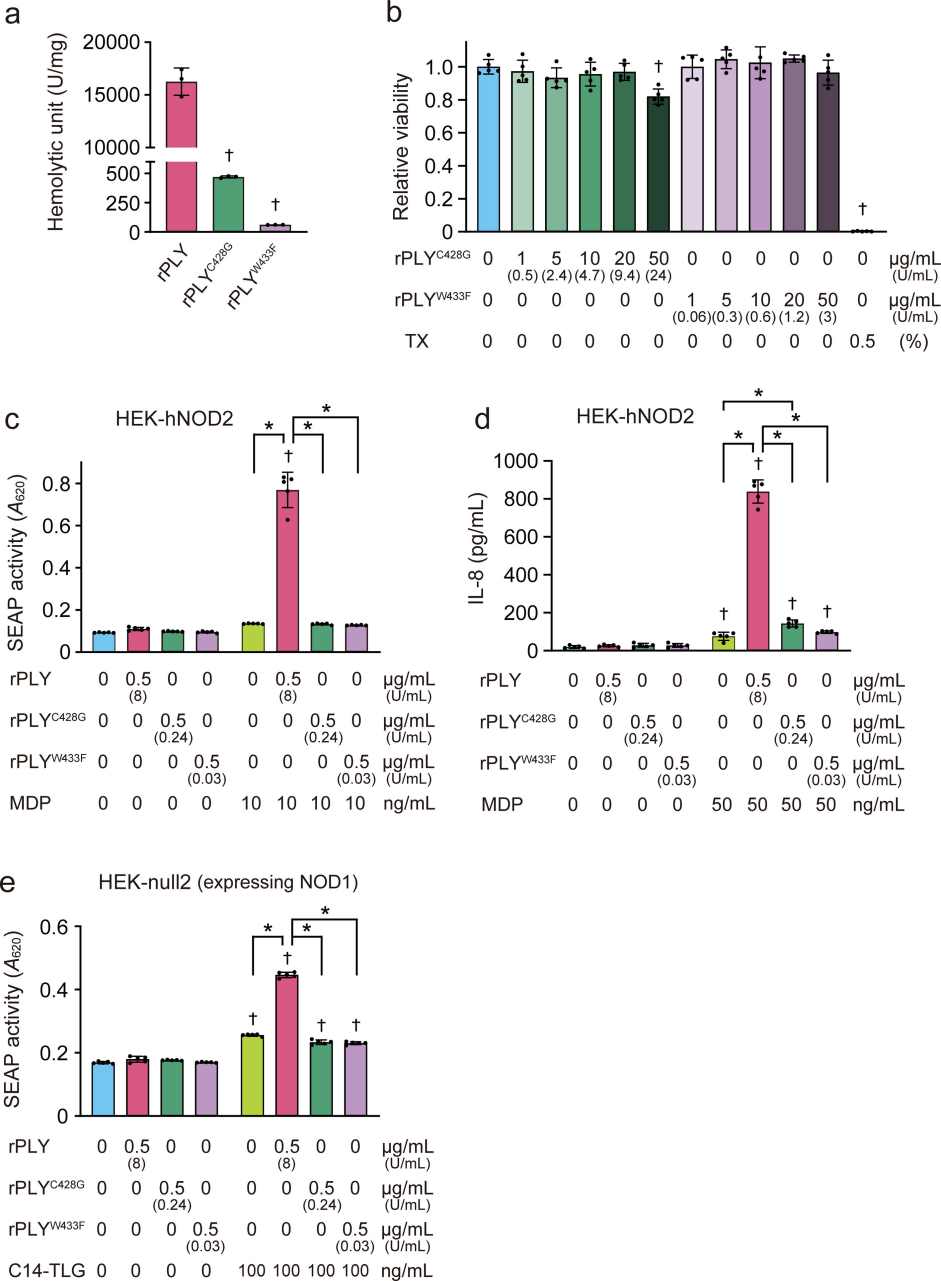
Pore-forming PLY significantly enhances NOD1 ligand- and NOD2 ligand-induced NF-κB activation in HEK-Blue cells, whereas non-pore-forming rPLYs do not. (**a**) Hemolytic activities of recombinant pore-forming PLY (rPLY; 70–350 ng/mL) and derivatives of PLY with mutations C428G and W433F (rPLY^C428G^ [1–10 μg/mL] and rPLY^W433F^ [20–66 μg/mL]) were evaluated on sheep erythrocytes. (**b**) HEK-hNOD2 cells were exposed to various concentrations of rPLY^C428G^ or rPLY^W433F^ for 16 h, followed by an MTT cell viability assay. (**c and d**) HEK-hNOD2 cells were exposed to rPLY, rPLY^C428G^, or rPLY^W433F^ in the presence or absence of MDP for 14 and 24 h, followed by (**c**) SEAP activity assay and (**d**) IL-8 ELISA, respectively. (**e**) HEK-null2 cells were exposed to rPLY, rPLY^C428G^, or rPLY^W433F^ in the presence or absence of C14-TLG for 16 h, followed by SEAP activity assay. Data represent the mean ± SD of quintuplicate experiments and were evaluated using a one-way analysis of variance with (**a and b**) Dunnett’s or (**c–e**) Tukey’s multiple comparisons test. † indicates a significant difference compared with (**a**) rPLY group (bright red) or (**b–e**) the control group (light blue) at *P* < 0.05. * indicates a significant difference between the indicated groups at *P* < 0.05. C14-TLG, C14-Tri-LAN-Gly; MDP, muramyl dipeptide; MTT, 3-(4,5-dimethylthiazol-2-yl)-2,5-diphenyltetrazolium bromide; NOD, nucleotide-binding oligomerization domain-containing protein; rPLY, recombinant pneumolysin; SD, standard deviation; SEAP, secreted alkaline phosphatase; TX, Triton X-100.

**Fig 4:**
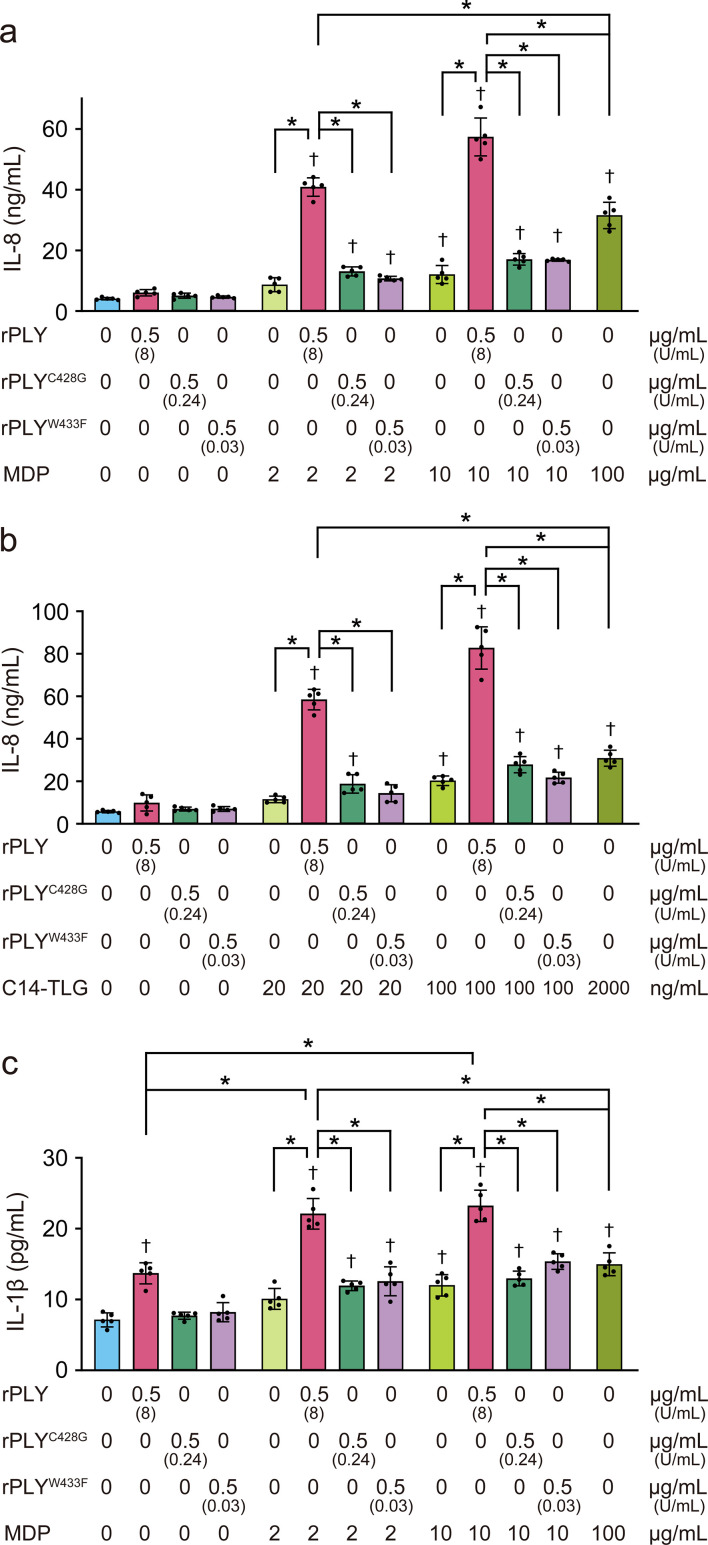
Pore-forming rPLY significantly enhances NOD1 ligand- and NOD2 ligand-induced IL-8 production in THP-1 cells, whereas non-pore-forming PLYs do not. PMA-differentiated THP-1 macrophages were exposed to rPLY, rPLY^C428G^, or rPLY^W433F^ in the presence or absence of (**a**) MDP or (**b**) C14-TLG for 24 h. IL-8 levels in the culture supernatants were measured using ELISA. (**c**) IL-1β levels in the culture supernatants of macrophages exposed to rPLYs and MDP were measured using ELISA. Data represent the mean ± SD of quintuplicate experiments and were evaluated using a one-way analysis of variance with Tukey’s multiple comparisons test. † indicates a significant difference compared with the control group (light blue) at *P* < 0.05. * indicates a significant difference between the indicated groups at *P* < 0.05. C14-TLG, C14-Tri-LAN-Gly; MDP, muramyl dipeptide; PMA, phorbol 12-myristate 13-acetate; rPLY, recombinant pneumolysin; SD, standard deviation.

We hypothesized that the pore-forming activity of rPLY contributes to the enhancement of NOD1/NF-κB and NOD2/NF-κB signaling. Thus, we used two derivatives of rPLY with C428G (rPLY^C428G^) and W433F mutations (rPLY^W433F^), which reportedly show reduced hemolytic activity compared to rPLY (28). Figure 3a shows that rPLY^C428G^ and rPLY^W433F^ retain approximately 3% and 0.3% of the hemolytic activity of rPLY. Figure 3b shows that 50 μg/mL rPLY^C428G^ exhibited slight cytotoxicity toward HEK293 cells, whereas ≤20 µg/mL rPLY^C428G^ did not. Additionally, ≤50 µg/mL rPLY^W433F^ did not show significant cytotoxicity toward the cells. We next exposed HEK-hNOD2 cells to 0.5 μg/mL rPLY, rPLY^C428G^, or rPLY^W433F^ in the presence or absence of NOD ligands. Figure 3c and d show that pore-forming rPLY significantly enhanced MDP-induced production of SEAP and IL-8, whereas neither rPLY^C428G^ nor rPLY^W433F^ enhanced their production. Additionally, neither rPLY^C428G^ nor rPLY^W433F^ enhanced SEAP production in HEK-null cells stimulated with C14-TLG (Fig. 3e). Furthermore, the synergetic effects of pore-forming rPLY and NOD ligands were observed in THP-1 macrophages, as rPLY significantly enhanced IL-8 production in the culture supernatants of both MDP- and C14-TLG-stimulated THP-1 macrophages (Fig. 4a and b). PLY has been shown to induce IL-1β production in an NLRP3 inflammasome-dependent manner (19). Therefore, we evaluated IL-1β levels in the supernatant of THP-1 macrophages exposed to rPLYs in the presence or absence of MDP. In contrast to the findings regarding IL-8 in Fig. 4a and b, which were not significantly increased in THP-1 macrophages upon stimulation with rPLY alone, pore-forming rPLY alone significantly increased IL-1β production by THP-1 macrophages (Fig. 4c), consistent with a previous report (19). Additionally, rPLY enhanced MDP-induced production of IL-1β.

### PLY is associated with increased NOD2 activation by pneumococcal culture supernatant

**Fig 5:**
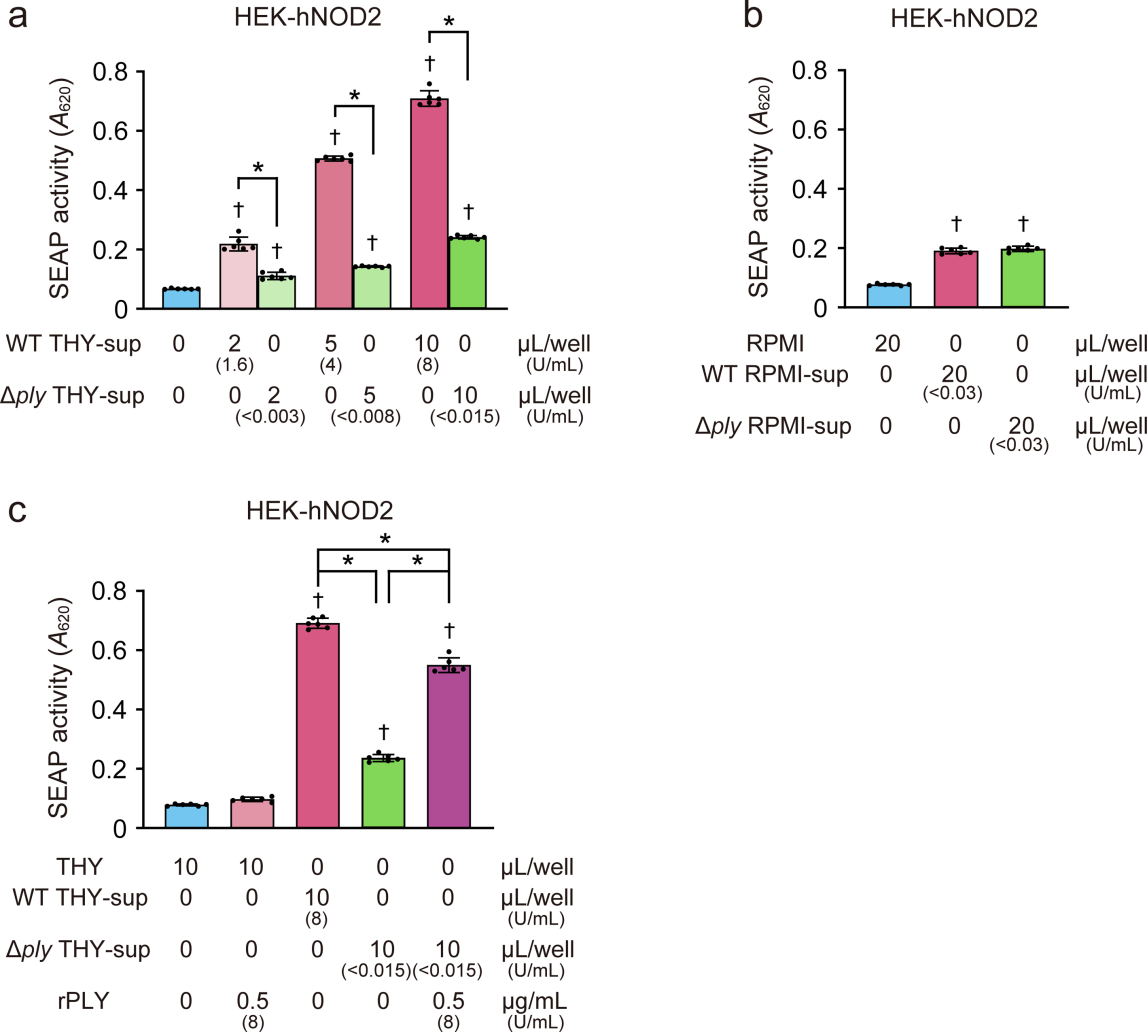
Culture supernatant from wild-type *S. pneumoniae* strain induces higher levels of NOD2 activation than that from Δ*ply*. (**a and b**) HEK-hNOD2 cells were incubated with culture supernatant (sup) from *S. pneumoniae* wild-type strain D39 (WT) or Δ*ply*, which was cultured in (**a**) THY extract broth or (**b**) RPMI 1640 supplemented with 10% fetal bovine serum for 14 h, followed by SEAP activity assay. (**c**) HEK-hNOD2 cells were incubated with culture supernatant from Δ*ply*, which was cultured in THY, in the presence or absence of rPLY for 14 h, followed by SEAP activity assay. Data represent the mean ± SD of sextuplicate experiments and were evaluated using a one-way analysis of variance with Tukey’s multiple comparisons test. † indicates a significant difference compared with the control group (light blue) at *P* < 0.05. * indicates a significant difference between the indicated groups at *P* < 0.05. hNOD2, human nucleotide-binding oligomerization domain-containing protein 2; rPLY, recombinant pneumolysin; SD, standard deviation; SEAP, secreted alkaline phosphatase; sup, supernatant; THY, Todd Hewitt yeast extract broth.

Pneumococcal peptidoglycan has been reported to induce NOD2 activation (8). Our previous study also demonstrated that pneumococcal supernatant induces NOD2-dependent NF-κB activation in HEK-hNOD2 cells (29). The results from this and previous studies suggest that *S. pneumoniae* may enhance the inflammation induced by its peptidoglycan through the action of PLY. To validate this, HEK-hNOD2 cells were incubated with the pneumococcal supernatant from the WT strain or Δ*ply*. Figure 5a shows that the culture supernatant of WT induced significantly higher SEAP activity compared to that of Δ*ply*. Previously, we have reported that *S. pneumoniae* cultured in RPMI 1640 medium supplemented with 10% bovine serum (RPMI) lacks hemolytic activity, thereby implying reduced levels of PLY, which we speculate could be attributable to a suppression of pneumococcal autolysis (29). To analyze the effect of PLY on pneumococcal supernatant-induced NF-κB activation in HEK-hNOD2 cells, we cultured pneumococcal strains in RPMI. Figure S3 shows that the culture supernatant of the WT strain exhibited hemolytic activity when cultured in Todd Hewitt yeast extract broth (THY) but not when cultured in RPMI, suggesting a lack of PLY in the RPMI-based pneumococcal supernatants. We then added these RPMI-based supernatants to the HEK-hNOD2 cell culture. As a result, SEAP levels in HEK-hNOD2 cells stimulated with the RPMI-based culture supernatant of WT became comparable to those induced in the cells by the supernatant of Δ*ply* (Fig. 5b). Furthermore, rPLY significantly increased SEAP levels in HEK-hNOD2 cells stimulated with THY-based culture supernatant of Δ*ply* compared to those in the cells stimulated with THY-based culture supernatant of Δ*ply* alone (Fig. 5c). These findings support the hypothesis that PLY enhances peptidoglycan-induced inflammation via NOD2 and reinforces its proinflammatory role in pneumococcal disease.

### IL-8 production was significantly decreased in THP-1 macrophages stimulated with culture supernatant of WT *S. pneumoniae* treated with a NOD2 selective inhibitor

**Fig 6:**
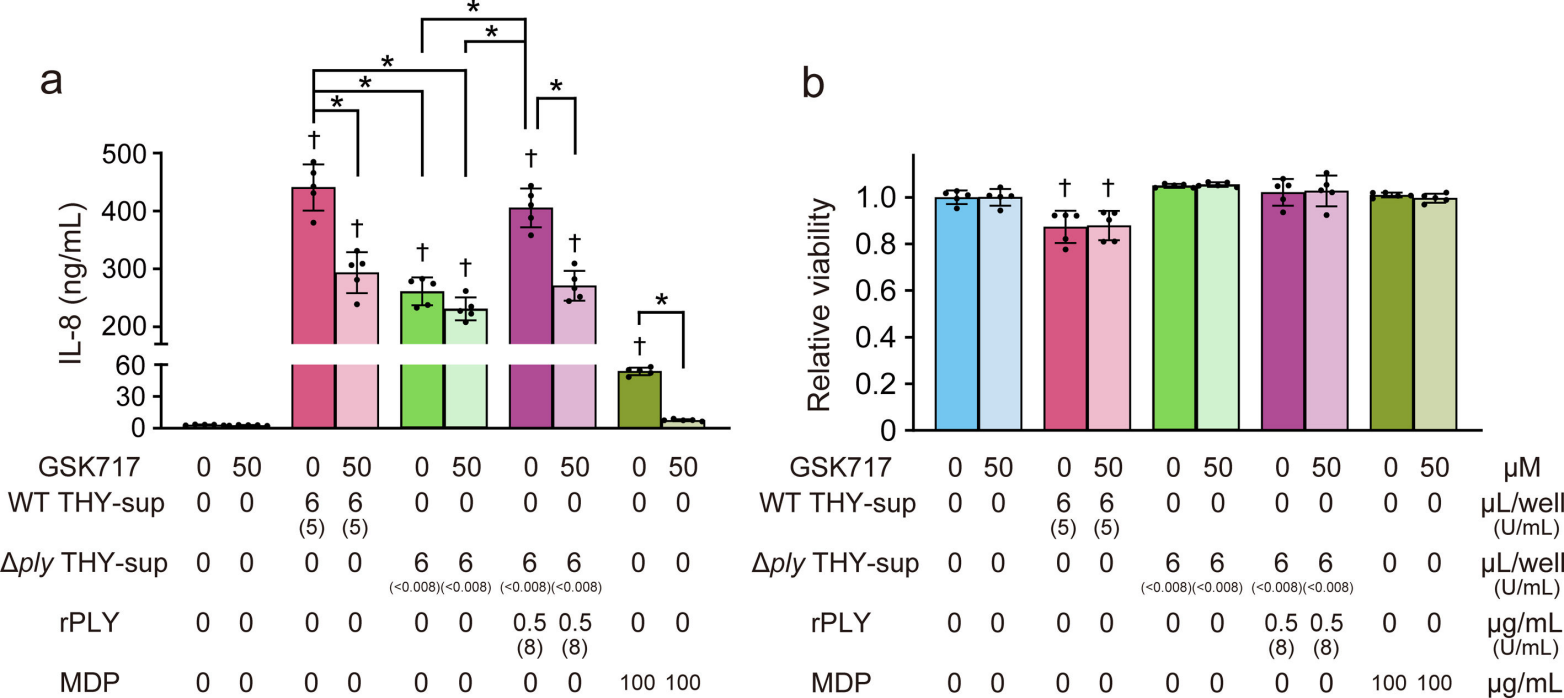
NOD2 inhibitor significantly decreased IL-8 production in THP-1 macrophages stimulated with the culture supernatant of wild-type *S. pneumoniae*. (**a and b**) PMA-differentiated THP-1 macrophages were exposed to culture supernatant (sup) from *S. pneumoniae* wild-type strain D39 (WT) or Δ*ply*, which was cultured in THY extract broth, in the presence or absence of NOD2-selective inhibitor GSK717 for 24 h. (**a**) IL-8 levels in the culture supernatant were measured using ELISA. (**b**) Cell viability was assessed using the MTT assay. Data represent the mean ± SD of quintuplicate experiments and were evaluated using a one-way analysis of variance with Tukey’s multiple comparisons test. † indicates a significant difference compared with the untreated control group at *P* < 0.05. * indicates a significant difference between the indicated groups at *P* < 0.05. MDP, muramyl dipeptide; PMA, phorbol 12-myristate 13-acetate; SD, standard deviation.

Although PLY promoted *S. pneumoniae* induced-NOD2 activation, TLR2 has been shown to play a central role in the innate immune recognition of Gram-positive bacteria (30). In our previous study, we demonstrated that the culture supernatant of *S. pneumoniae* activates both TLR2 and NOD2 (29). To clarify the role of PLY-enhanced NOD2 activation in the innate immune recognition of *S. pneumoniae*, we treated THP-1 macrophages with GSK717, a selective NOD2 inhibitor, which blocks NOD2-mediated IL-8 production but does not inhibit TLR2- or NOD1-mediated IL-8 production (31). Figure 6a shows that GSK717 decreased MDP-induced IL-8 production by 85% in THP-1 macrophages. Consistent with Fig. 5a, IL-8 levels were significantly lower in cells stimulated with Δ*ply* culture supernatant than those stimulated with WT culture supernatant. Additionally, co-treatment of THP-1 macrophages with rPLY and the Δ*ply* culture supernatant resulted in significantly higher IL-8 production than treatment with the Δ*ply* supernatant alone. Furthermore, GSK717 significantly reduced IL-8 production under both stimulation conditions—with WT culture supernatant or with a mixture of rPLY and Δ*ply* culture supernatants—to levels comparable to those observed in the cells stimulated with the Δ*ply* culture supernatant alone. Figure 6b shows that 6 μL WT culture supernatant slightly decreased the cell viability. GSK717 did not significantly affect the WT culture supernatant-mediated cytotoxicity, suggesting that GSK717 does not inhibit PLY-mediated pore formation. These findings suggest that TLR2 is the predominant receptor mediating IL-8 production in response to *S. pneumoniae*. In addition, NOD2 appears to substantially contribute to IL-8 production in a manner dependent on PLY-mediated pore formation.

### Pore-forming rPLY facilitates entry of extracellular molecules into host cell cytoplasm

**Fig 7:**
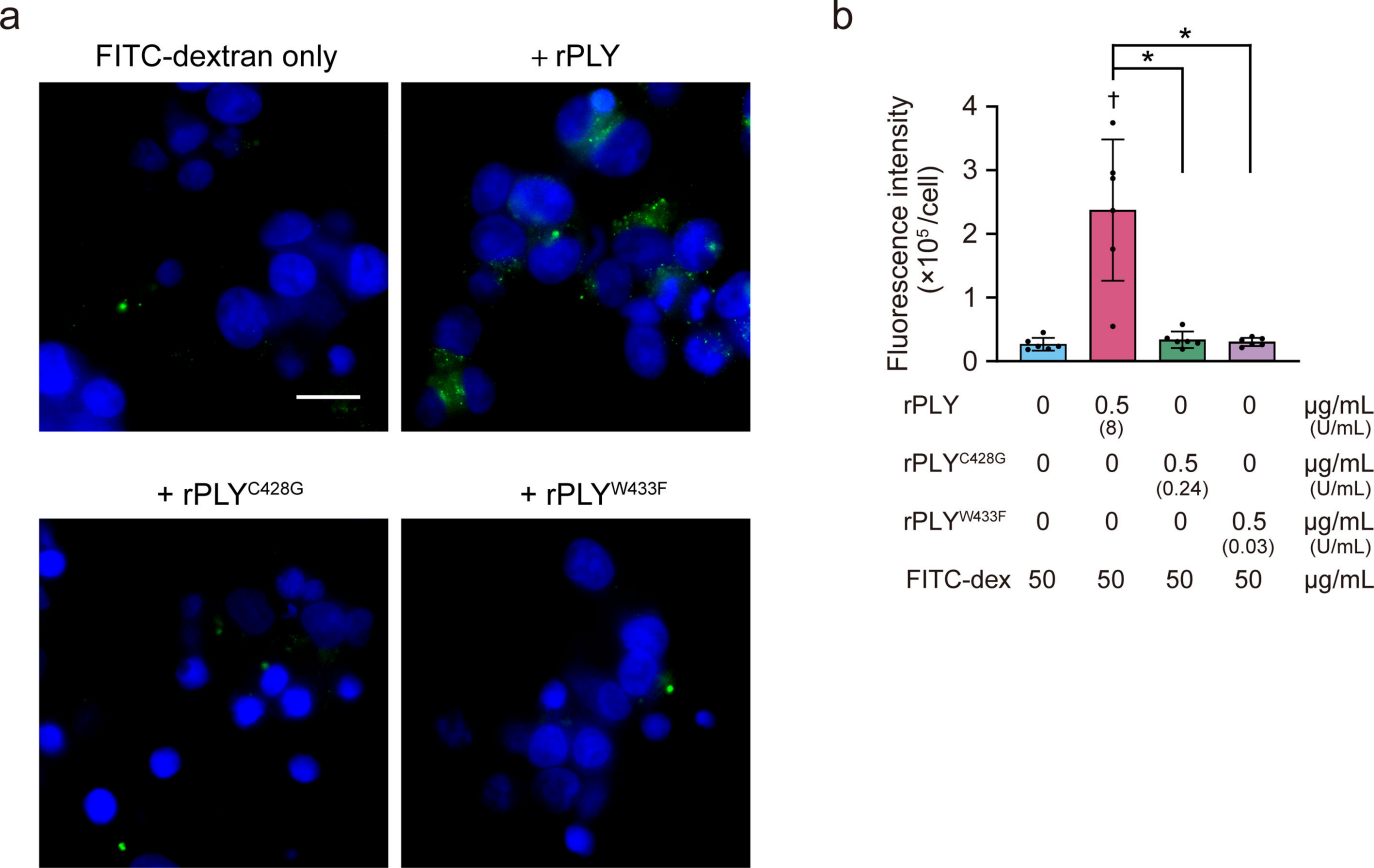
Pore-forming rPLY triggers the influx of fluorescently labeled dextran into the cytoplasm of HEK293 cells, whereas non-pore-forming PLYs do not. HEK-hNOD2 cells were exposed to rPLY, rPLY^C428G^, or rPLY^W433F^ in the presence of FITC-dextran (green) for 24 h, followed by nucleic acid staining (DAPI; blue). (**a**) Representative images obtained by fluorescence microscopy are shown. Scale bar: 20 μm. (**b**) The fluorescence intensity of FITC per cell was calculated. Data represent the mean ± SD of six randomly selected images from triplicate experiments and were evaluated using a one-way analysis of variance with Tukey’s multiple comparisons test. † indicates a significant difference compared with the control group (light blue) at *P* < 0.05. * indicates a significant difference between the indicated groups at *P* < 0.05.

NOD1 and NOD2 sense peptidoglycans internalized from bacteria (26). Therefore, we hypothesized that rPLY-induced pore formation in the host cell membrane results in an influx of extracellular molecules, including peptidoglycans, into the cells. To test this hypothesis, we exposed HEK-hNOD2 cells to rPLY, rPLY^C428G^, or rPLY^W433F^ in the presence of FITC-labeled dextran (4 kDa), followed by fluorescence microscopy. Figure 7a and b show that rPLY significantly induced the intracellular accumulation of FITC-dextran compared to FITC-dextran alone, rPLY^C428G^, or rPLY^W433F^.

## DISCUSSION

**Fig 8:**
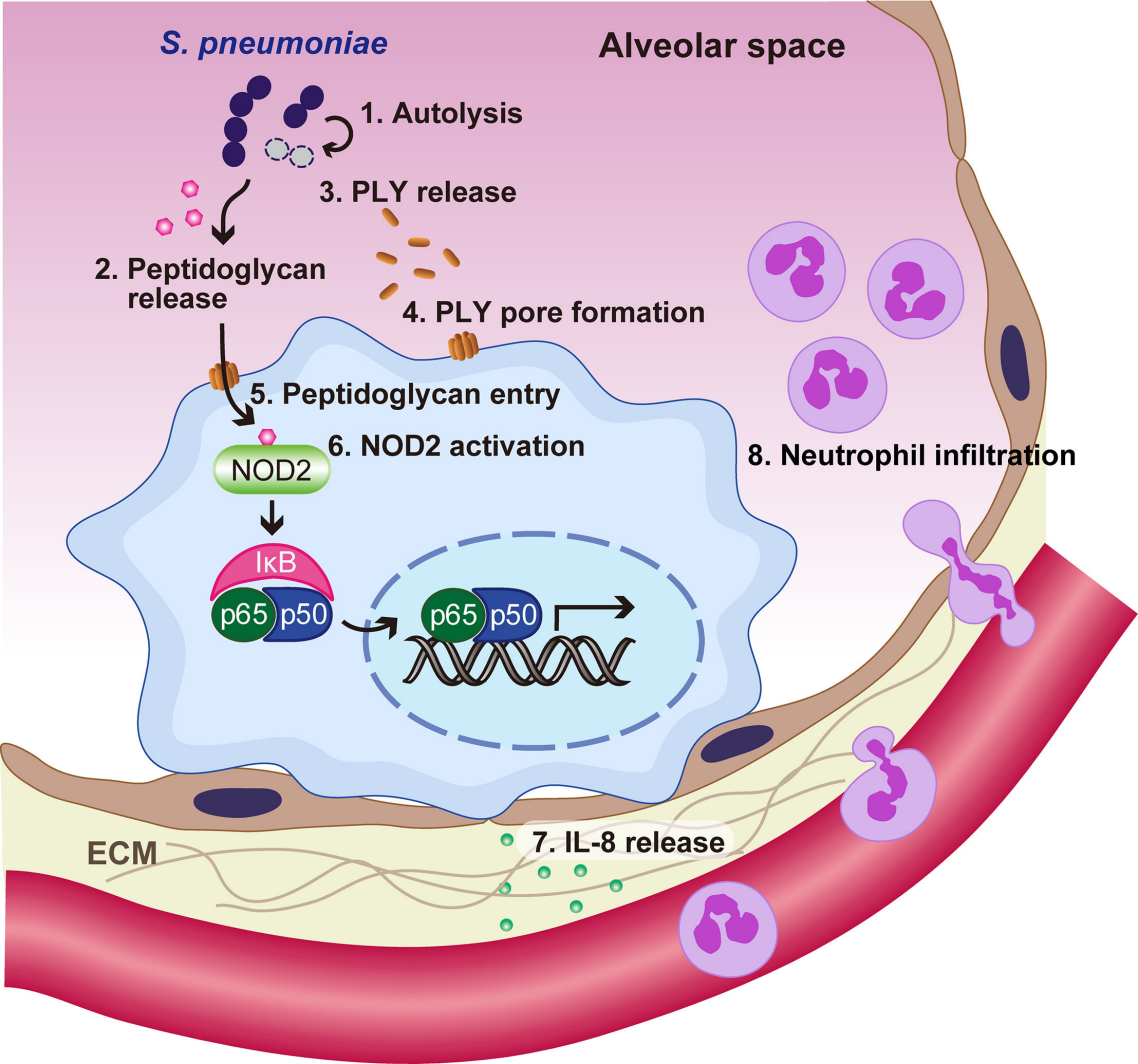
Summary of PLY-mediated enhancement of NOD2 signaling by *S. pneumoniae*. Upon colonization or infection, *S. pneumoniae* (1) undergoes autolysis, leading to the release of (2) innate immune stimulants, such as peptidoglycan, and (3) pneumolysin (PLY), which (4) form pores in the host cell membrane. This leads to (5) an influx of peptidoglycan into the cytoplasm through these pores, resulting in (6) activation of the NOD2 cytoplasmic peptidoglycan sensor. This activation (7) induces the production of cytokines, such as IL-8, leading to (8) the recruitment of neutrophils.

A previous study reported that PLY-induced pores allow the capsular polysaccharide, a pneumococcal surface component, to access the host cell cytosol (32). Additionally, the pore-forming properties of PLY enhanced *H. influenzae* peptidoglycan-induced NOD1 activation (24). As pneumococcal peptidoglycan induces NOD2-dependent signaling, we hypothesized that PLY contributes to this process (8). To test this hypothesis, we treated HEK-hNOD2 cells with rPLY and its derivatives, which showed significantly decreased pore-forming activity. We demonstrated that rPLY does not directly activate TLR4 or the other PRRs examined in this study, but instead enhances cytosolic receptor NOD2 signaling. Additionally, rPLY enhanced C14-TLG-induced NOD1/NF-κB activation, which is consistent with previous findings. Given that the enhancement of NOD signaling was induced exclusively by pore-forming rPLY, the underlying mechanism appears to involve membrane pores formed by rPLY, which facilitate the influx of extracellular molecules, such as pneumococcal peptidoglycan, into the cell, leading to the activation of NOD signaling (Fig. 8). These findings provide new insights into the contribution of PLY to the pathogenesis of pneumonia.

NOD1 and NOD2 are members of the cytosolic NOD-like receptor family and recognize conserved motifs in bacterial peptidoglycans, leading to the activation of the NF-κB and MAPK pathways and subsequent production of proinflammatory responses. NOD1 recognizes γ-D-glutamyl-meso-diaminopimelic acid that is found in the peptidoglycan of all Gram-negative bacteria (33), whereas NOD2 senses MDP, a peptidoglycan motif common to both Gram-positive and Gram-negative bacteria (25). Although pneumococcal peptidoglycan is recognized by NOD2, the role of NOD2 in pneumococcal diseases remains controversial. Hommes et al. reported that NOD2-deficient mice were indistinguishable from wild-type mice with regard to pneumococcal load in the lungs during pneumococcal pneumonia and concluded that NOD2 does not play a significant role in host defense during the disease (34). However, Davis et al. reported that Nod2 and TLR2 contribute to the clearance of pneumococcal colonization, as mice lacking both receptors (*Tlr2^–/–^Nod2^–/–^*) showed a marked decrease in chemokine expression and impaired bacterial clearance compared with WT and single-knockout mice, indicating the importance of Nod2 in effective pneumococcal clearance (8). These findings also suggest that TLR2 and Nod2 have redundant roles, since elimination of an individual receptor was not associated with a measurable defect in pneumococcal clearance. Wang et al. reported that the NOD2 signaling pathway is involved in microglial inflammation via the NF-κB pathway and neutrophil infiltration into meningeal tissue in a mouse pneumococcal meningitis model (35). Although neutrophils are required for the clearance of pathogenic bacteria, including *S. pneumoniae*, excessive recruitment of neutrophils results in tissue damage owing to the release of neutrophil proteases (36, 37). We previously demonstrated that PLY preferentially targets neutrophils over alveolar epithelial cells and macrophages, resulting in neutrophil death and subsequent protease release (15). Additionally, even sublytic concentrations of PLY stimulate neutrophils to form neutrophil extracellular traps, which contain high levels of proteases (38). Taken together, the promotion of NOD2 signaling by PLY may have a beneficial role in promoting pneumococcal clearance by neutrophils. However, it may also have a detrimental effect by promoting neutrophil-mediated tissue damage. Further research is required to determine the role of NOD2 signaling in pneumococcal diseases.

The potential recognition of PLY by TLR4 was first reported by Malley et al. in 2003 (9). A subsequent study also demonstrated that PLY induces tumor necrosis factor-α via TLR4 in murine RAW 264.7 macrophages (11). However, McNeela et al. demonstrated that endotoxin-free rPLY activates the NLRP3 inflammasome and induces cytokine secretion in murine dendritic cells in a TLR4-independent manner (19). Consistent with this, pore-forming PLY induces the production of IL-1β in murine and human mononuclear cells in an NLRP3-dependent manner, whereas PLY with reduced hemolytic activity does not (20). One possible mechanism for PLY-induced NLRP3 activation involves K^+^ efflux through the pore structure (19, 39). In contrast, Chiu et al. recently reported that both full-length rPLY and rPLY domain 4, which is responsible for pore formation, activate secretion of proinflammatory cytokines in murine bone marrow dendritic cells in a TLR4-dependent manner (10). However, the non-pore-forming rPLY domain 1-3 protein failed to do so (10). Therefore, PLY-induced pores may contribute to the indirect activation of TLR4. It has been speculated that TLR4 recognizes damage-associated molecular patterns (DAMPs) released following PLY-induced host cell damage (18). Indeed, at a lytic concentration of 2 μg/mL, rPLY induced the release of high mobility group box 1 (HMGB1), a DAMP that acts as an endogenous TLR4 ligand, into the culture supernatant of HEK-null2 cells (Fig. S4). Collectively, we propose that PLY, rather than being directly recognized by PRRs, induces pores that serve as channels for the influx of peptidoglycan into the cytosol, induces K^+^ efflux, and triggers the release of DAMPs, leading to the subsequent activation of NODs, NLRP3, and TLR4, respectively.

During pore formation, the PLY monomer binds to the host cell membrane and oligomerizes to form a pre-pore complex. In this step, the tryptophan-rich loop of PLY domain 4 binds to cholesterol in the cell membrane, whereas domains 1–3 remain extracellular and contribute to the formation of a pre-pore complex (40). Thereafter, the pre-pore complex undergoes structural changes to form a transmembrane pore composed of 30–50 subunits (41). The transmembrane pore is 32–43 nm in diameter (40). Although the diameter of a typical protein can vary depending on its structure, it has been reported that the diameter of a single globular protein molecule is approximately <10 nm (42, 43). Therefore, transmembrane pores may facilitate the bidirectional transport of bacterial and host-derived proteins, such as peptidoglycan and DAMPs, across the cell membrane.

It has been reported that in alveolar macrophages, PLY binds to mannose receptor C type 1 (MRC-1) (44), thus indicating that receptor-mediated host responses may also contribute to pneumococcal pathogenesis. However, although PLY–MRC-1 interactions may contribute to the responses observed in THP-1 macrophages, we also consistently detected a PLY-mediated enhancement of NOD activation in HEK-Blue cells, which are generally considered to express minimal levels of MRC-1 (45). These findings would thus tend to indicate that rather than MRC-1-dependent signaling, the formation of membrane pores is the primary mechanism underlying PLY-enhanced NOD activation.

TLR3 and TLR9 are members of nucleic acid-sensing TLRs that are expressed in endosomes. The activation of these receptors is preceded by ligand endocytosis and delivery into endosomes (46). In this study, rPLY enhanced NODs signaling but had minimal impact on poly(I:C)-induced TLR3 activation and CpG oligodeoxynucleotide 2006-induced TLR9 activation. These findings suggest that the membrane pores formed by PLY allow extracellular molecules to directly access the cytosol but do not enhance endocytosis in HEK293 cells. However, Hupp et al. have reported that PLY-induced pores accelerate endocytosis in glial cells (47). The differences in these data may be attributed to variations in the rPLY concentrations and cell types. Further investigation is required to elucidate the effects of PLY on endocytic events.

This study is limited in that the use of HEK reporter cells and excess rPLY or NOD ligands may not fully reflect the physiological interactions between *S. pneumoniae* and primary innate immune cells. Although our data support a model in which PLY forms the host plasma membrane pores to facilitate the influx of NOD ligands, it remains unclear whether this mechanism operates *in vivo*, as it would require sufficient ligand shedding under physiological conditions. In addition, because NOD2 expression in the alveolar space is largely restricted to alveolar macrophages rather than alveolar epithelial cells, we used THP-1 macrophages to model this cellular context. However, THP-1 cells do not fully recapitulate the phenotype or functional responses of primary alveolar macrophages, representing a limitation of our study.

PLY is released during LytA-mediated pneumococcal autolysis (13), and a *lytA* mutant strain induces minimal inflammation in the mouse model of intranasal infection (48), suggesting that PLY likely acts extracellularly. However, PLY-induced pores in the host phagosomal membrane have also been reported to facilitate the release of pneumococcal components, including peptidoglycan, into the host cell cytosol, resulting in NOD2 activation (8). The independent contributions of PLY-induced pore formation in the plasma membrane versus the phagosome to NOD2 activation remain unresolved. Further experiments are therefore needed to clarify the relative roles of plasma membrane- and phagosome-associated PLY pores in immune cell activation *in vivo*.

In conclusion, our results suggest that PLY-induced pores contribute to the proinflammatory activity of *S. pneumoniae* by activating the cytosolic receptor NOD2. Although this study focused on the effect of PLY on TLR- and NOD-mediated innate immune responses owing to the limited repertoire of PRR-expressing HEK-Blue cell lines, several other cytosolic PRRs have also been identified. For example, RIG-I-like receptors detect viral RNA, while absent in melanoma 2 recognizes microbial DNA, including that of *S. pneumoniae*. Cyclic GMP-AMP synthase plays a crucial role in host defense by sensing viral, bacterial, and host-derived DNA (49). Thus, PLY may enhance the activation of these cytosolic receptors. Furthermore, pore-forming toxins, such as PLY, may serve as valuable tools in innate immunology research, potentially enabling the identification of novel cytosolic receptor ligands.

## MATERIALS AND METHODS

### Reagents

rPLY, rPLY^C428G^, and rPLY^W433F^ were expressed in *Escherichia coli* and purified as previously described (15, 50). The rPLYs were dialyzed against phosphate-buffered saline (PBS). Thereafter, endotoxin in the samples was depleted using a ToxinEraser endotoxin removal kit (GenScript, Piscataway Township, NJ, USA), and the concentration of LPS in 1 μg of rPLY proteins was determined to be <1 pg using an LPS-detection kit (GenScript).

### LPL treatment for lipoprotein digestion

rPLY, rPLY^C428G^, and rPLY^W433F^ were treated with LPL from *Burkholderia* sp. (Sigma-Aldrich, Inc., St. Louis, MO, USA), as previously described, with modifications (23). Briefly, these recombinant proteins (30 μg/mL) were incubated with 10 mg/mL LPL in PBS at 37°C for 14 h. We also confirmed that LPL did not induce SEAP production in HEK-hTLR2 cells.

### Hemolytic activity assay

The hemolytic activities of rPLY, rPLY^C428G^, and rPLY^W433F^ were evaluated as previously described (51). Briefly, a 1% suspension of sheep erythrocytes (Japan BioSerum, Hiroshima, Japan) in PBS was exposed to various concentrations of rPLY (70–350 ng/mL), rPLY^C428G^ (1–10 μg/mL), or rPLY^W433F^ (20–66 μg/mL) and incubated at 37°C for 30 min, followed by centrifugation at 2,000 × *g* for 7 min. The absorbance of the supernatant was measured at 571 nm using a Nivo S multimode plate reader (PerkinElmer Japan G.K., Kanagawa, Japan). A hemolytic unit is the concentration that leads to 50% lysis of sheep erythrocytes (52).

### Preparation of pneumococcal culture supernatants

*S. pneumoniae* wild-type strain D39 or Δ*ply* was grown in THY (Becton Dickinson, Franklin Lakes, NJ, USA) supplemented with 0.5% yeast extract (Becton Dickinson) under aerobic conditions at 37°C. After an overnight incubation, the cultures were inoculated into a freshly prepared THY or RPMI (RPMI 1640 medium [FUJIFILM Wako Pure Chemical Corporation, Osaka, Japan] supplemented with 10% fetal bovine serum [FBS]) and cultured until they reached the stationary phase (OD_600_ of approximately 0.8 and 0.3 in THY and RPMI, respectively). The pneumococcal culture supernatants were collected by centrifugation (5,000 × *g* for 10 min at 37°C) and filtration through a 0.2-μm pore size filter (Sartorius Stedim Biotech GmbH, Göttingen, Germany), and used for SEAP activity assay using HEK-Blue cells.

### SEAP activity assay

HEK-Blue hTLR2, hTLR4, hTLR9, hNOD2, and null2 cells were purchased from InvivoGen (San Diego, CA, USA). These cells were grown in Dulbecco’s modified Eagle’s medium (DMEM; FUJIFILM Wako Pure Chemical Corporation) supplemented with 10% FBS, 1% penicillin-streptomycin solution (FUJIFILM Wako Pure Chemical Corporation), 100 μg/mL Normocin (InvivoGen), and corresponding selective antibiotics at 37°C in a humidified atmosphere with 5% CO_2_. These cell lines were used between passages 5 and 10. After proliferation, cells were suspended in HEK-Blue detection media (InvivoGen), plated into a 96-well plate at a density of 5 × 10^4^ cells per 200 μL, and stimulated with 2–20 μL pneumococcal supernatant, 2 ng/mL Pam3CSK4 (InvivoGen), 50–200 ng/mL poly(I:C) (InvivoGen), 1–10 ng/mL LPS from *E. coli* 055:B5 (Sigma-Aldrich, Inc.), 0.8–2 µg/mL CpG oligodeoxynucleotide 2006 (InvivoGen), 100–300 ng/mL C14-TLG (InvivoGen), 10–100 ng/mL MDP (InvivoGen), or 1–5 µg/mL PGN-SA (InvivoGen) in the presence or absence of 0.5 μg/mL rPLY for 14–18 h. Given that the kinetics of ligand-induced SEAP production differ slightly among the different pattern recognition receptors, we optimized the incubation times for each of the different HEK-Blue cell lines. SEAP activity in the culture supernatant was measured using a microplate reader (PerkinElmer Japan G.K.) at 620 nm.

### Cell culture and stimulation

THP-1 cells (RIKEN cell bank, Tsukuba, Japan) were grown in RPMI 1640 medium supplemented with 10% FBS, 1% penicillin-streptomycin solution at 37°C in a humidified atmosphere with 5% CO_2_. The cells were used between passages 10 and 15. The cells were activated in a 96-well culture plate at a density of 1 × 10^5^ cells per 200 μL in the medium supplemented with 200 nM phorbol 12-myristate 13-acetate (Cayman Chemical Company, Ann Arbor, MI, USA) to induce their differentiation into macrophage-like cells. After 48 h, the cells were washed extensively with fresh RPMI 1640 medium and cultured for 12 h. Thereafter, the cells were stimulated with 20–2,000 ng/mL C14-TLG or 2–100 μg/mL MDP in the presence or absence of 0.5 μg/mL rPLY for 24 h. Regarding the NOD2 inhibition experiments, THP-1 macrophages were preincubated with 50 μM GSK717 (Merck KGaA, Darmstadt, Germany) for 1 h, followed by stimulation with THY-based pneumococcal culture supernatants (6 μL/well) or 100 μg/mL MDP in the presence or absence of 0.5 μg/mL rPLY for 24 h.

HEK-hNOD2 cells were suspended in DMEM supplemented with 10% FBS, 1% penicillin-streptomycin solution, 100 μg/mL Normocin, plated into a 96-well plate at a density of 1 × 10^5^ cells per 200 μL, and stimulated with 50 ng/mL MDP in the presence or absence of 0.5 μg/mL rPLY for 24 h.

### Cytokine assay

The levels of IL-8 and IL-1β in the cell culture supernatants of THP-1 or HEK-hNOD2 cells were measured using ELISA kits (BioLegend, San Diego, CA, USA) according to the manufacturer’s instructions.

### Cell viability assay

HEK-Blue cells were incubated in a 96-well culture plate at a density of 1 × 10^5^ cells per 200 μL in DMEM supplemented with 10% FBS, 1% penicillin-streptomycin solution, 100 μg/mL Normocin at 37°C in a humidified atmosphere with 5% CO_2_. The cells were exposed to various concentrations of rPLY, rPLY^C428G^, rPLY^W433F^, or pneumococcal supernatants for 16 h. THP-1 macrophages (1 × 10^5^ cells/200 μL) were exposed to pneumococcal supernatants or MDP in the presence or absence of GSK717 for 24 h. Thereafter, 50 μL of 3-(4,5-dimethylthiazol-2-yl)-2,5-diphenyltetrazolium bromide (MTT; 2 mg/mL in PBS; Dojindo Laboratories Co., Ltd., Kumamoto, Japan) was added to each well, followed by incubation for 2 h. The supernatant was carefully removed by aspiration. Formazan product formed in the cells was dissolved in 100 μL acidified isopropanol, and the absorbance was measured at 540 nm using a Multimode Plate Reader (PerkinElmer Japan G.K.).

### Assay for intracellular uptake of FITC-dextran

HEK-hNOD2 cells were incubated in a glass-bottom multiwell dish (Matsunami Glass Ind., Ltd., Osaka, Japan) at a density of 1 × 10^5^ cells per 200 μL in DMEM supplemented with 10% FBS, 1% penicillin-streptomycin solution, 100 μg/mL Normocin at 37°C in a humidified atmosphere with 5% CO_2_. Thereafter, the cells were exposed to 0.5 μg/mL rPLY, rPLY^C428G^, or rPLY^W433F^ in the presence of 50 μg/mL FITC-dextran (4 kDa; TdB Labs, Uppsala, Sweden) for 24 h, followed by fixation with 10% formalin neutral buffer (FUJIFILM Wako Pure Chemical Corporation) and nucleic acid staining with 4′,6-diamidino-2-phenylindole dihydrochloride (DAPI; Dojindo Laboratories Co., Ltd.). Fluorescent images were captured using a BIOREVO BZ-9000 microscope (Keyence, Osaka, Japan). The fluorescence intensity of FITC per cell was calculated using a Hybrid Cell Count Application (Keyence).

### Statistics and reproducibility

Sample sizes for each experiment are described in the corresponding figure legend. Data were analyzed by one-way analysis of variance with Dunnett’s or Tukey’s multiple comparison test using GraphPad Prism version 9.5.1 (GraphPad Software, Inc., La Jolla, CA, USA). Data were presented as mean ± standard deviation values from at least three replicates.

## Data Availability

All data described are contained within the manuscript and supplemental material.

## SUPPLEMENTAL MATERIAL

Supplemental figures spectrum.00249-26-s0001.docx Fig. S1 to S4.

ASM does not own the copyrights to Supplemental Material that may be linked to, or accessed through, an article. The authors have granted ASM a non-exclusive, world-wide license to publish the Supplemental Material files. Please contact the corresponding author directly for reuse.
