# Supplementary material for: Extracellular pneumolysin enhances the activation of cytosolic pattern-recognition receptor NOD2 through its pore-forming activity
Source: Microbiol Spectr. 2026 Jul 14;14(8):e00249-26. doi: 10.1128/spectrum.00249-26 (PMC13435831; doi:10.1128/spectrum.00249-26)
Supplement: Supplementary file 1 [file spectrum.00249-26-s0001.docx]

**Supplementary Information**

Extracellular pneumolysin enhances the activation of cytosolic pattern-recognition receptor NOD2 through its pore-forming activity

Hisanori Domon^1,2^, Satoru Hirayama^1^, Toshihito Isono^1^, Tomoki Maekawa^2^, Yutaka Terao^1,2*^

^1^Division of Microbiology and Infectious Diseases, Niigata University Graduate School of Medicine, Dentistry and Health Sciences, Niigata, Japan

^2^Center for Advanced Oral Science, Niigata University Graduate School of Medicine, Dentistry and Health Sciences, Niigata, Japan

*Correspondence:

Yutaka Terao

terao@dent.niigata-u.ac.jp

**Supplementary Fig. 1: Lipoprotein lipase-treated recombinant PLY induced significantly lower SEAP levels in HEK-hTLR2 cells than untreated recombinant PLY.**

To digest contaminated lipopeptides/lipoproteins, rPLY and Pam3CSK4 (Pam3; a synthetic triacylated lipopeptide used as a control) were treated with lipoprotein lipase (LPL) at 37°C for 12 h. Thereafter, HEK-hTLR2 cells were stimulated with these samples for 14 h, followed by SEAP activity assay. Data represent the mean ± SD of quintuplicate experiments and were evaluated using one-way analysis of variance (ANOVA) with Tukey’s multiple comparison test. † indicates significant difference compared with the control group (light blue) at *P* < 0.05. * indicates significant difference between the indicated groups at *P* < 0.05.

HEK-hTLR2, HEK-Blue cells expressing human TLR2; human Toll-like receptor 2; rPLY, recombinant pneumolysin; SD, standard deviation; SEAP, secreted alkaline phosphatase.

**Supplementary Fig. 2:** **Neither TLR stimulants alone nor in combination with recombinant PLY leads to a significant increase in SEAP activity in HEK-null2 cells.**

HEK-null2 negative control cells were exposed to rPLY in the presence or absence of: **a** lipopolysaccharide (LPS), **b** Pam3CSK4 (Pam3), **c** muramyl dipeptide (MDP), **d** peptidoglycan from *Staphylococcus aureus* (PGN-SA), or **e** CpG oligodeoxynucleotide 2006 (ODN) for 16–18 h, followed by SEAP activity assay. Data represent the mean ± SD of sextuplicate experiments and were evaluated using a one-way analysis of variance with Tukey’s multiple comparisons test. † indicates significant difference compared with the control group (light blue) at *P* < 0.05. * indicates significant difference between the indicated groups at *P* < 0.05.

rPLY, recombinant pneumolysin; SD, standard deviation; SEAP, secreted alkaline phosphatase

**Supplementary Fig. 3: Pneumococcal culture supernatant does not exhibit hemolytic activity when cultured in RPMI 1640 medium supplemented with 10% bovine serum.**

Wild-type *Streptococcus pneumoniae* strain D39 (WT) and a *ply*-isogenic mutant strain (Δ*ply*) were grown in Todd Hewitt yeast extract broth (THY) and RPMI 1640 medium supplemented with 10% fetal bovine serum (RPMI) until they reached the stationary phase of growth (OD_600_ of approximately 0.8 and 0.3 in THY and RPMI, respectively). Subsequently, the hemolytic activity of the culture supernatants (sup) was evaluated in sheep erythrocytes. Data represent the mean ± SD of quintuplicate experiments. ND indicates below the detection limit of 0.3 U/mL.

ND, not detected; SD, standard deviation

**Supplementary Fig. 4: rPLY-induced cell death results in the release of** **high mobility group box 1 protein into the culture supernatant of HEK-null2 cells.**

HEK-null2 cells (1 × 10^6^ cells) were exposed to 2 μg/mL rPLY in Dulbecco’s modified Eagle’s medium supplemented with 10% FBS for 16 h at 37°C. High mobility group box 1 protein (HMGB1) levels in 15 μL of culture supernatant were determined by Western blotting using an anti-HMGB1 monoclonal antibody (Abcam, Cambridge, UK). rPLY-untreated HEK-null2 cell lysate was used as a positive control. Representative Western blot images are shown.
